# A meta-analysis of efficacy on dexamethasone and clomiphene in the treatment of polycystic ovary syndrome patients

**DOI:** 10.1186/s12905-024-03141-9

**Published:** 2024-05-20

**Authors:** Ying Sha, Jianfen Zhu, Fangrong Shen

**Affiliations:** 1 Department of Obstetrics and Gynecology, Zhangjiagang Second People’s Hospital, Zhangjiagang City, Jiangsu Province China; 2 Department of Obstetrics and Gynecology, The First Affiliated Hospital of Suzhou University, Suzhou City, Jiangsu Province China

**Keywords:** Polycystic ovary syndrome, Dexamethasone, Clomiphene, Meta-analysis

## Abstract

**Objective:**

Polycystic ovary syndrome (PCOS) is an endocrine gynecological disease affecting many women of reproductive age. Clomiphene is the first-line treatment for PCOS patients, but most individuals may be resistant to it. This study aims to assess the efficacy of dexamethasone and clomiphene in the treatment of PCOS patients, and to provide a theoretical basis for clinicians to study and treat PCOS.

**Methods:**

Chinese and English databases including PubMed, Embase, Cochrane Library, China National Knowledge Infrastructure (CNKI), WanFang Medical Network, and VIP Information Chinese Journal Service Platform (VIP) were searched from the inception to January 2023. Review Manager and Stata software were used for meta- analysis. The risk of bias of eligible studies were assessed using Cochrane’s risk of bias tool. Publication bias was assessed by funnel plots, Begg’s and Egger’s tests.

**Results:**

A total of 12 literatures were finally included, with a total of 1270 PCOS patients. Compared with the control group, dexamethasone combined with clomiphene could significantly improve pregnancy (*RR* = 1.71, *P* < 0.00001), ovulation (*RR* = 1.30, *P* < 0.00001), luteinizing hormone level (SMD = -0.94, *P* < 0.00001), estradiol level (SMD = 0.99, *P* = 0.05), progesterone level (SMD = 5.08, *P* = 0.002) and testosterone level (SMD = -1.59, *P* < 0.00001). However, there were no significant effects on ovulation-stimulating hormone level (SMD = 0.15, *P* = 0.37), adverse reactions (*RR* = 1.30, *P* = 0.30), dizziness (*RR* = 1.50, *P* = 0.45), and vomiting (*RR* = 1.67, *P* = 0.48).

**Conclusion:**

The treatment of dexamethasone combined with clomiphene is helpful to improve the ovulation and pregnancy rate in patients with PCOS, and improve the hormone levels of patients.

## Introduction

Polycystic ovary syndrome (PCOS) is an endocrine and gynecological disease that affects many women of reproductive age. Patients will experience symptoms of endocrine disorders, including irregular secretion of follicle-stimulating hormone (FSH) and luteinizing hormone, and significant insulin resistance [1]. PCOS is primarily caused by an elevated ratio of luteinizing hormone (LH) to FSH and an increased release of gonadotropin-releasing hormone. However, its exact etiology is complex, involving genetic, environmental, and lifestyle factors. There is still controversy over its exact etiology and pathology [2, 3].

Some studies have shown that clomiphene citrate is considered as the primary ovulation-inducing medication for infertile PCOS patients. Clomiphene citrate is an anti-estrogen therapy that blocks estrogen receptors in the hypothalamus, and stimulates follicular development through a negative feedback mechanism [4, 5]. Dexamethasone can inhibit the biological activity of aromatase and at the same time regulate the level of estrogen. Previous studies have shown that a short course of dexamethasone treatment can lead to a significant increase in FSH levels in female rats [6, 7]. Guan Qiong et al. have reported that dexamethasone combined with clomiphene played an important role in improving serum hormones, which reduced serum LH and testosterone (T) levels, while increasing estradiol and FSH levels [8].

Previous studies have shown that dexamethasone combined with clomiphene may improve pregnancy and ovulation outcomes in patients with PCOS. But there is no clear evidence. Therefore, this study used meta-analysis to comprehensively and systematically assess the efficacy and safety of dexamethasone combined with clomiphene therapy in patients with PCOS.

## Methods

### Literature search strategy

Based on the Cochrane Handbook guidelines, this research conducted a search of both Chinese and English databases including PubMed, Embase, Cochrane Library, China National Knowledge Infrastructure (CNKI), WanFang Medical Network, VIP Information Chinese Journal Service Platform (VIP), and the retrieval time was from inception to January 2023. The search keywords in Chinese and English were “Dexamethasone”, “Clomifene”, “Clomiphene”, “Polycystic ovary syndrome” and so on. The retrieval languages mainly included Chinese and English, and other languages were excluded in this study.

### Eligibility criteria

In this study, only randomized controlled trials (RCTs) determining the effects of combining dexamethasone with clomiphene for PCOS patients were included. In these studies, the observation group was PCOS patients treated with dexamethasone combined with clomiphene. The control group was treated with dexamethasone or clomiphene; the study did not limit the dose or route of administration. This study excluded animal experimental studies, prospective studies, cohort experimental studies, reviews, and other types of literatures.

### Data extraction

The information required for the study from the included research literature were independently extracted by two researchers. The data included the author’s name, publication year, geographical location, sample size of intervention and control groups, treatment measures in the intervention and control groups, and main outcome data. The third researcher conducted information verification and data proofreading to ensure the accuracy.

### Quality assessment

Two researchers independently assessed the quality of the included studies using the Cochrane risk of bias assessment tool [9]. The instrument was comprised of the subsequent components: (1) Selection bias; (2) Allocation blinding; (3) Blinding of investigators and participants; (4) Blind assessment of research findings; (5) Completeness of outcome data; (6) Selective reporting of research findings; (7) Other sources of bias. The risk of bias was classified as low, high or unclear levels. Results were illustrated using various color blocks, accompanied by corresponding risk of bias maps. If there was any discrepancy, the final evaluation would rely on the findings of the third researcher.

### Statistical analysis

For continuous variables, we utilized the standardized mean difference (SMD) and its 95% CI. For dichotomous variables, we utilized the relative risk (RR) and its 95% CI. Study heterogeneity was evaluated using the I^2^ statistic and the χ^2^ test. If there was substantial heterogeneity, subgroup analyses and sensitivity analyses were used to investigate the underlying reasons. We evaluated publication bias by employing both Begg’s test and Egger’s test. The forest plots and funnel charts were created using Review Manager 5.3 software. STATA 15.1 software was used to conduct statistical analysis. Statistically significant differences were determined at a significance level of *P* < 0.05.

## Results

### Literature search results

**Fig. 1:**
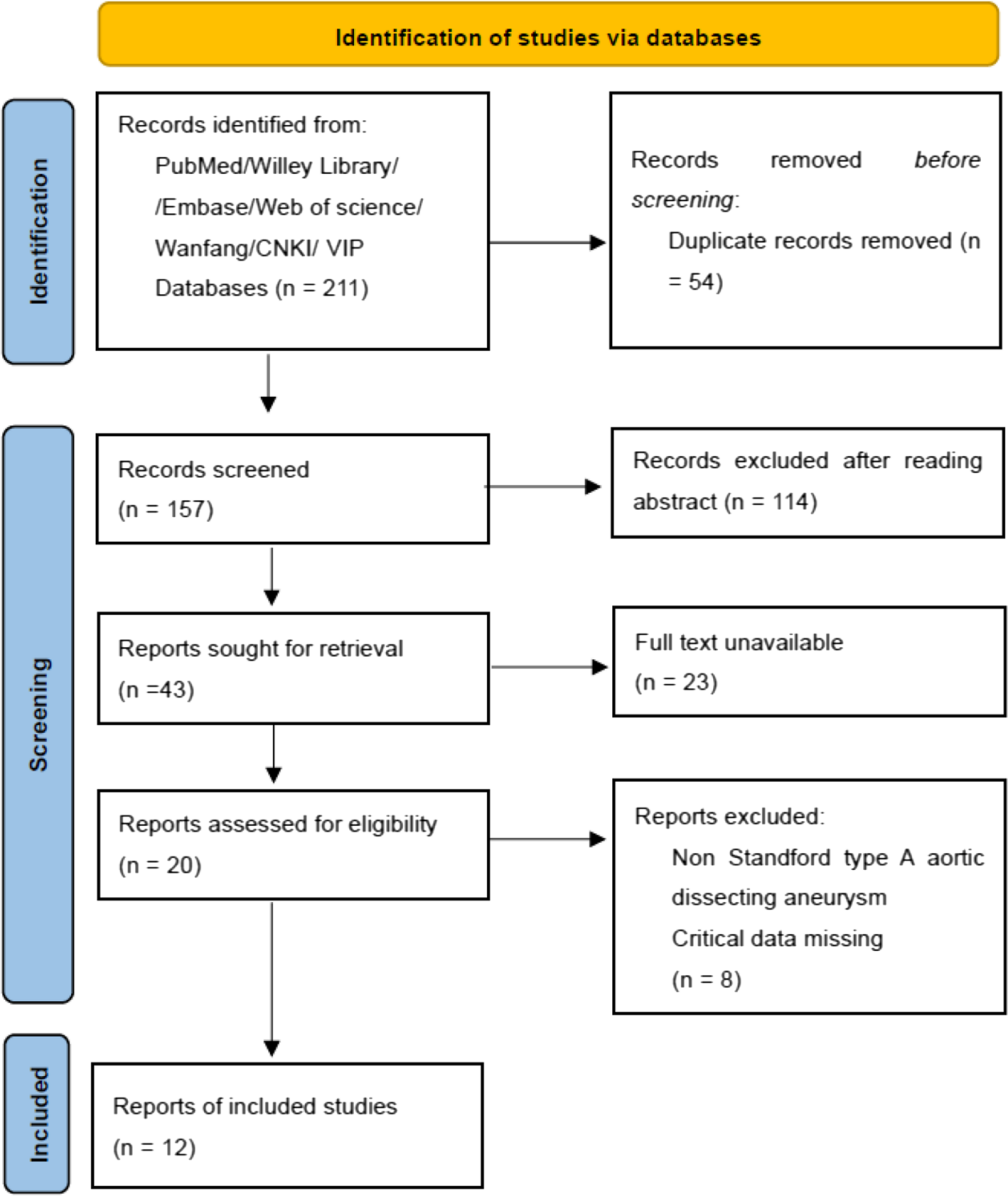
Document screening flow chart

Literature retrieval was carried out according to the established retrieval strategy. A total of 211 literatures were retrieved initially in this study, and 170 literatures were obtained after deduplication. 114 literatures were excluded by screening titles and abstracts. After full-text assessment of 20 related studies, we included 12 RCTs finally. The process of the literature screening was shown in Fig. 1.

### Basic characteristics of included studies

This study included a total of 12 clinical studies [8, 10–20]. Table 1 showed the fundamental features of the studies included.

**Table 1:** Basic characteristics of the included studies

Author	Country	Sample size		Treatment		Age		Course of disease	
		Treatment group	Control group	Treatment group	Control group	Treatment group	Control group	Treatment group	Control group
Qiong Guan, 2022 [8]	China	42	42	Clomiphene citrate capsules combined with dexamethasone acetate tablets 0.75 mg/dose, 1x/d, discontinued after ovulation	Dexamethasone acetate tablets, 50 mg/dose, 1x/d for 5d	29.1 ± 1.3	28.9 ± 1.6	3.3 ± 0.6	3.2 ± 0.5
Jingju Zheng, 2020 [10]	China	54	54	Dexamethasone combined with clomiphene, 0.75 mg/dose, 1x/d, 15d consecutive oral doses	Clomiphene, 50 mg/d for 5d	29.58 ± 1.84	29.64 ± 1.86	1.92 ± 0.36	1.89 ± 0.34
Yanhua Chen, 2019 [11]	China	68	62	Dexamethasone acetate tablets combined with clomiphene citrate, 2 mg/dose, 1x/d, 7d consecutive oral doses	Clomiphene citrate tablets, 100 mg/d for 7d	27.2 ± 5.0	27.6 ± 4.4	3.9 ± 0.4	3.7 ± 0.6
Wei Ma, 2018 [12]	China	54	54	Dexamethasone tablets combined with clomiphene tablets, 0.75 mg/dose, 1x/d, discontinued after ovulation	Clomiphene tablets, 50 mg/d for 5d	27.46 ± 3.42	28.11 ± 4.01	4.01 ± 0.23	3.03 ± 1.42
Peinign Huang, 2011 [13]	China	26	26	Dexamethasone combined with clomiphene tablets, 0.75 mg/dose, 1x/d, discontinued after ovulation	Clomiphene tablets, 50 mg/d for 5d				
Yuemei Lin, 2019 [14]	China	60	60	Dexamethasone in combination with clomiphene capsules 2 mg once daily for 8d	Clomiphene capsules, 100 mg once/d for 5d	33.28 ± 7.36	33.59 ± 7.14	3.35 ± 0.46	3.42 ± 0.51
Huina Wu, 2020 [15]	China	31	31	Clomiphene citrate capsules combined with dexamethasone acetate tablets, 1.5 mg/dose, 3 times/d for 3 weeks	Dexamethasone acetate tablets, 50 mg/d for 5d	29.52 ± 6.35	29.65 ± 6.21		
Shanshan Wang, 2018 [16]	China	51	51	Dexamethasone combined with clomiphene tablets, 0.75 mg/dose, 1x/d, 5d consecutive oral doses	Clomiphene tablets, 50 mg/d, 1x/d for 5d	28.12 ± 3.59	28.26 ± 3.67	2.31 ± 0.29	2.25 ± 0.27
Huimin Liu, 2020 [17]	China	47	47	Dexamethasone combined with clomiphene, 0.75 mg/dose, 1x/d, 5d consecutive oral doses	Clomiphene, 50 mg/d, 1x/d for 5d	31.42 ± 2.16	31.28 ± 2.12	2.31 ± 0.96	2.56 ± 1.05
Yunhua Wen, 2017 [18]	China	60	69	Dexamethasone tablets combined with clomiphene, 0.75 mg/dose, 1x/d, 5d continuous oral dose	Clomiphene, 50 mg/d, 1x/d for 5d	31.4 ± 7.3		2.45 ± 2.21	
Esmaeilzadeh S, 2011 [19]	Iran	30	30	Dexamethasone and clomiphene	Clomiphene and placebo	24.8 ± 3.56	23.1 ± 3.45	2.98 ± 1.85	3.15 ± 1.45
Parsanezhad ME, 2002 [20]	Iran	111	119	Dexamethasone combined with clomiphene, 2 mg/dose, 1x/d, 10d continuous oral dose	Clomiphene combined with placebo, 250 mg/d once/d for 5 d	23.56	23.36	4	4.25

### Risk of bias assessment

**Fig. 2:**
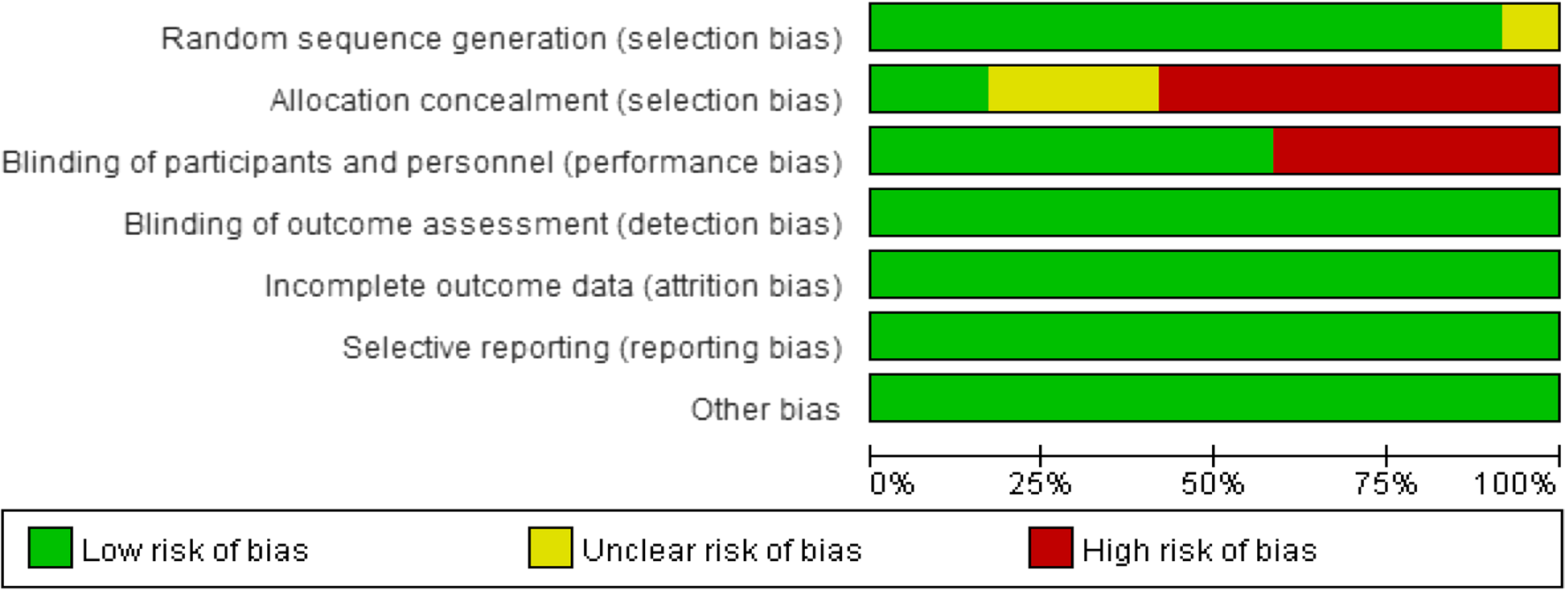
Risk of bias map

**Fig. 3:**
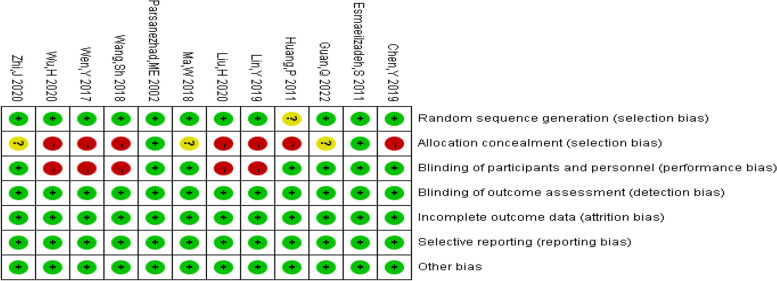
Overview of risk of bias

The risk of bias plots of the studies included were shown in Figs. 2 and 3. Most studies included in this study were at low risk of bias. Among them, 7 items contained high risk of bias scores, and no concealed groups was carried out. Five studies were not blinded.

### Results of meta-analyses

#### Effectiveness index

##### Pregnancy

**Fig. 4:**
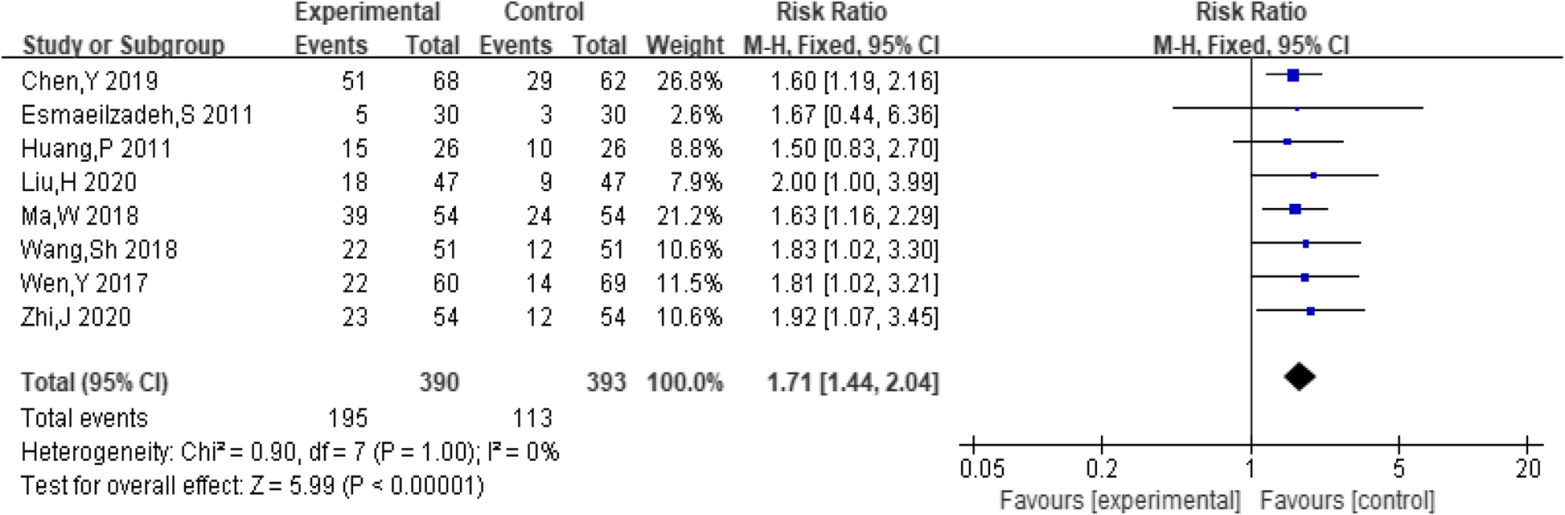
Forest plot of the effect of dexamethasone combined with clomiphene on pregnancy in patients with PCOS

**Fig. 5:**
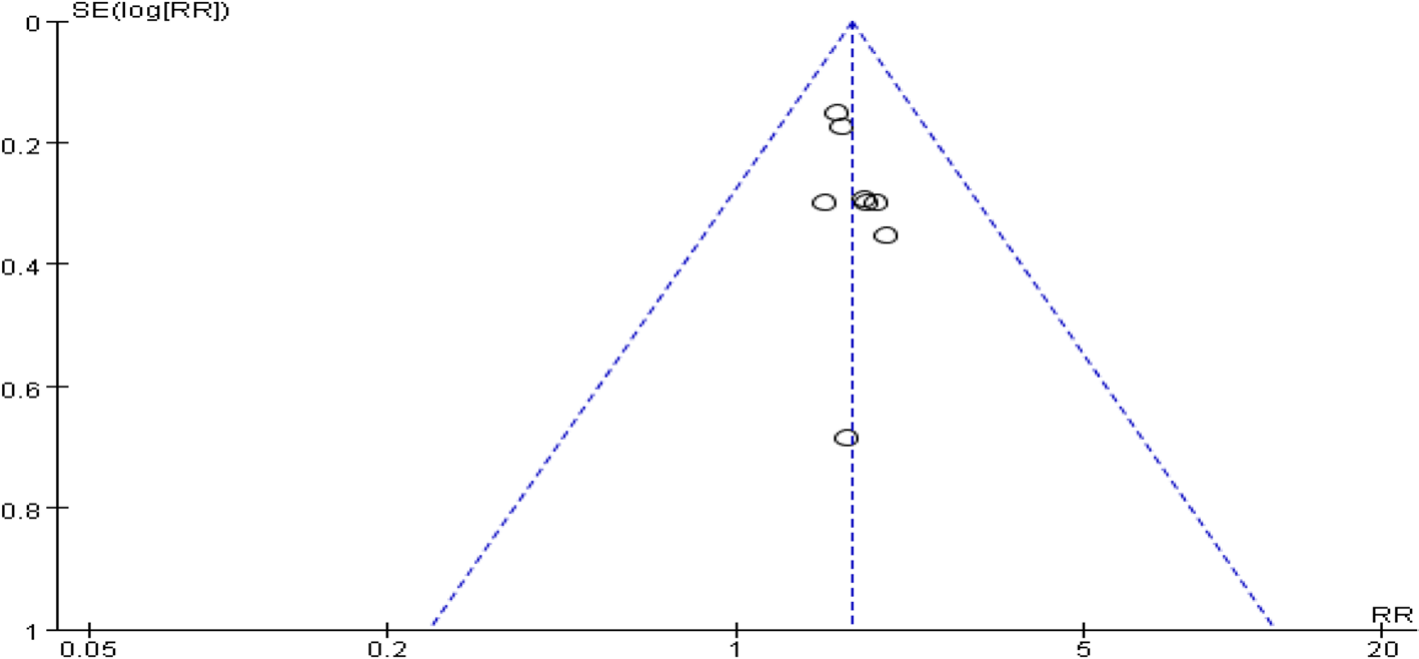
Funnel plot of the effect of dexamethasone combined with clomiphene on pregnancy in patients with PCOS

A total of 8 studies provided data on the number of pregnancies. The pooling data resulted in no heterogeneity among the studies (I^2^ = 0%, *P* = 1.00). Hence, the fixed-effect model was selected for analyzing this outcome. The results in Fig. 4 showed that the combination of dexamethasone and clomiphene could improve the pregnancy rate in patients with PCOS (*RR* = 1.71, 95% CI: 1.44, 2.04). Funnel plot, along with Begg’s and Egger’s tests indicated that there was no publication bias (Fig. 5).

##### Ovulation

**Fig. 6:**
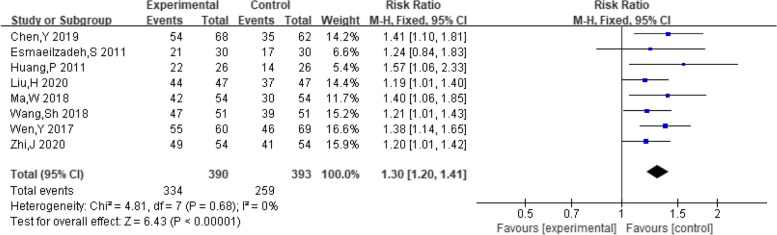
Forest plot of the effect of dexamethasone combined with clomiphene on ovulation in patients with PCOS

**Fig. 7:**
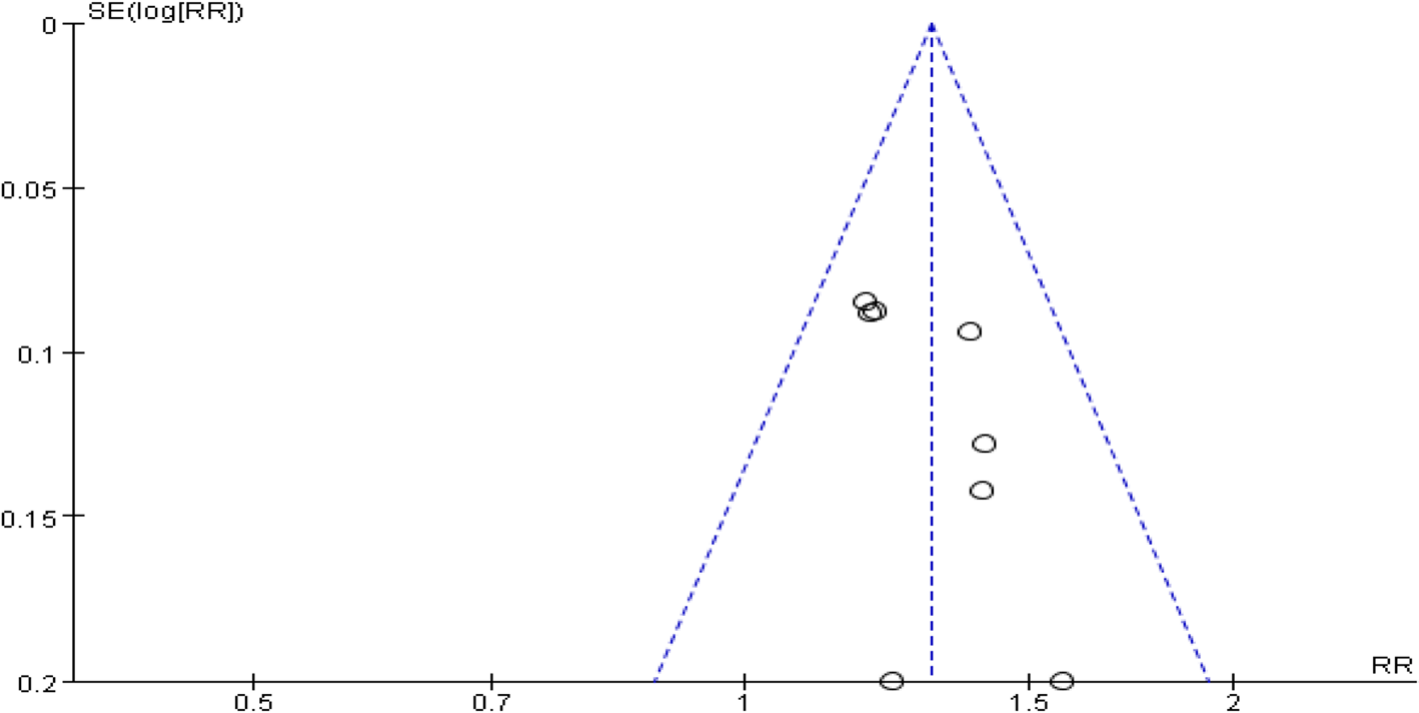
Funnel plot of the effect of dexamethasone combined with clomiphene on ovulation in patients with PCOS

A total of eight studies provided data on the number of female ovulations. The pooling data resulted in no heterogeneity among the studies (I^2^ = 0%, *P* = 0.68). Hence, the fixed-effect model was selected for analyzing this outcome. The results showed that dexamethasone combined with clomiphene treatment could improve the ovulation rate in individuals with PCOS (*RR* = 1.30, 95% CI: 1.20, 1.41, Fig. 6). Funnel plot, along with Begg’s and Egger’s tests indicated that there was no publication bias (Fig. 7).

##### Luteinizing hormone levels

**Fig. 8:**
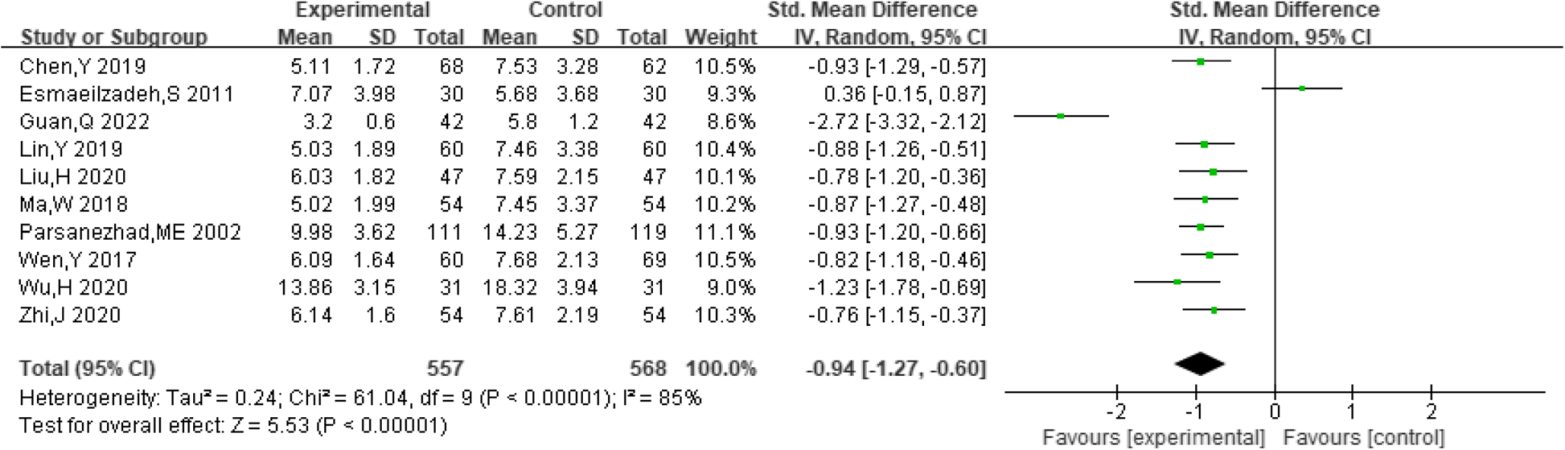
Forest plot of the effect of dexamethasone combined with clomiphene on the level of luteinizing hormone in patients with PCOS

**Fig. 9:**
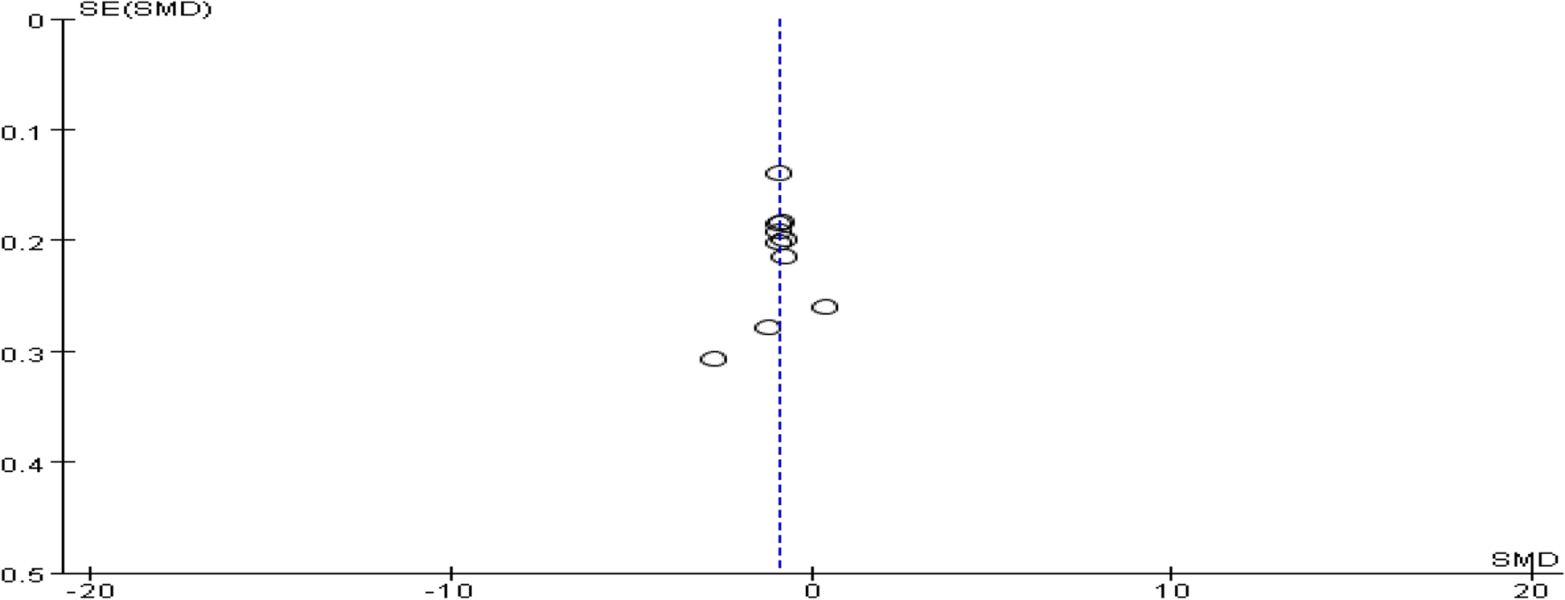
Funnel plot of the effect of dexamethasone combined with clomiphene on the level of luteinizing hormone in patients with PCOS

A total of ten studies provided data on patient luteinizing hormone levels. Heterogeneity among studies was found after pooling data (I^2^ = 85%, *P* < 0.00001). Hence, the random-effect model was selected for analyzing this outcome. The results indicated that the combination of dexamethasone and clomiphene could improve the level of luteinizing hormone in patients with PCOS (SMD = -0.94, 95% CI: -1.27, -0.60, Fig. 8). Funnel plot, along with Begg’s and Egger’s tests indicated that there was no publication bias (Fig. 9).

##### Ovulation-stimulating hormone level

**Fig. 10:**
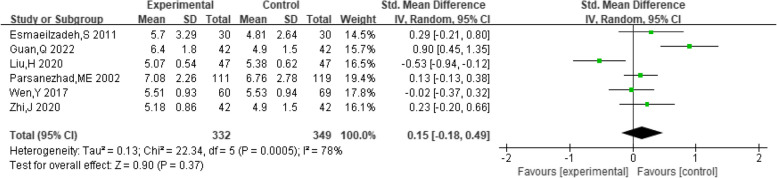
Forest plot of the effect of dexamethasone combined with clomiphene on the level of luteinizing hormone in patients with PCOS

A total of six studies provided data on the level of ovulation-stimulating hormone in patients. The pooling data showed substantial heterogeneity among the studies (I^2^ = 78%, *P* = 0.0005). Hence, a random-effect model was chosen to analyze this outcome. The findings indicated that the combination of dexamethasone and clomiphene had insignificant effect on enhancing levels of ovulation-stimulating hormone in PCOS patients (SMD = 0.15, 95% CI: -0.18, 0.49, Fig. 10).

##### Estradiol levels

**Fig. 11:**
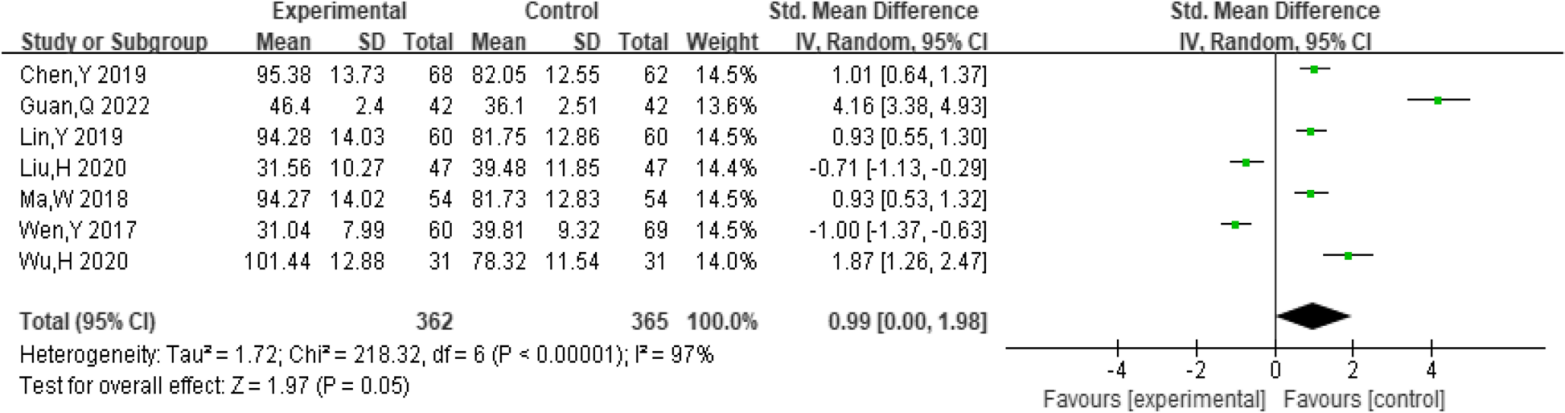
Forest plot of the effect of dexamethasone combined with clomiphene on the level of estradiol in patients with PCOS

A total of 7 studies provided data on patient estradiol levels. The pooling data showed substantial heterogeneity among the studies (I^2^ = 97%, *P* < 0.00001). Hence, a random-effect model was chosen to analyze this outcome. The findings indicated that the joint use of dexamethasone and clomiphene was effective in enhancing the estradiol levels in PCOS patients (SMD = 0.99, 95% CI: 0.00, 1.98, Fig. 11).

##### Progesterone levels

**Fig. 12:**
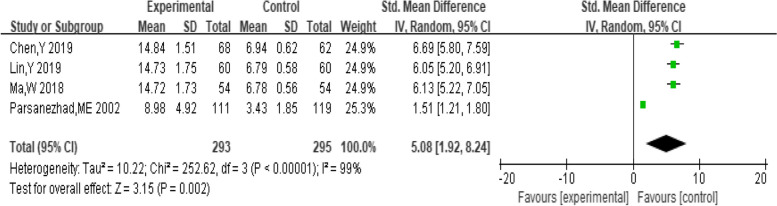
Forest plot of the effect of dexamethasone combined with clomiphene on progesterone levels in patients with PCOS

A total of four studies provided data on the progesterone levels of the patients. The pooling data showed substantial heterogeneity among the studies (I^2^ = 99%, *P* < 0.00001). Therefore, a random-effect model was selected to analyze this outcome. The results showed that dexamethasone combined with clomiphene treatment could improve progesterone levels in PCOS patients (SMD = 5.08, 95% CI: 1.92, 8.24, Fig. 12).

##### Testosterone levels

**Fig. 13:**
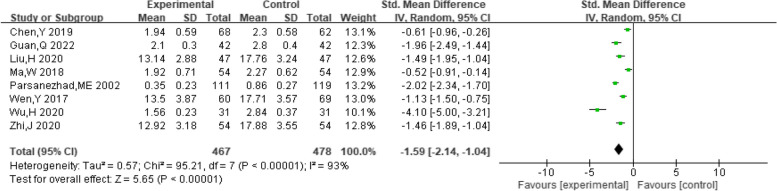
Forest plot of the effect of dexamethasone combined with clomiphene on testosterone levels in patients with PCOS

**Fig. 14:**
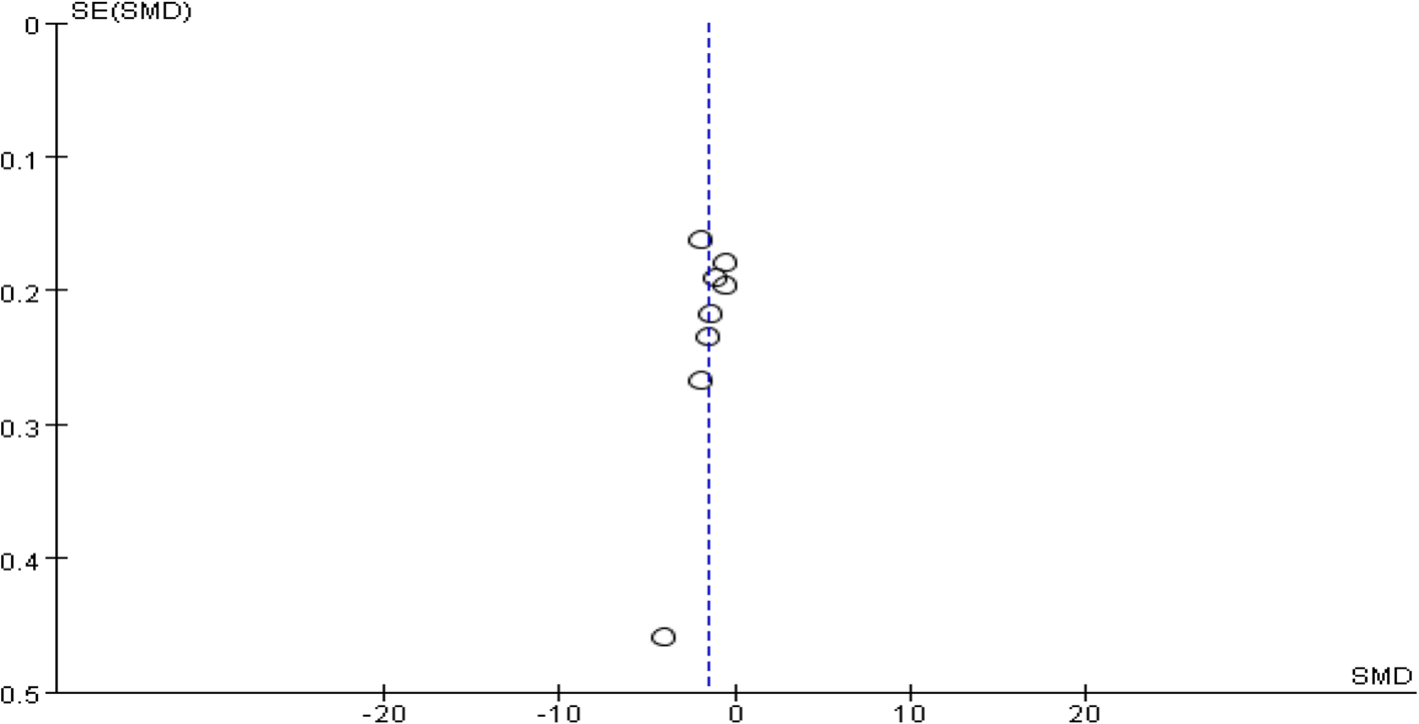
Funnel plot of the effect of dexamethasone combined with clomiphene on testosterone levels in patients with PCOS

A total of eight studies provided data on patient testosterone levels. Heterogeneity among studies was found after pooling data (I^2^ = 93%, *P* < 0.00001). Hence, a random-effect model was chosen to analyze this outcome. The results showed that dexamethasone combined with clomiphene treatment could improve testosterone levels in PCOS patients (SMD = -1.59, 95% CI: -2.14, -1.04, Fig. 13). Funnel plots, along with Begg’s and Egger’s tests indicated that there was no publication bias (Fig. 14).

#### Safety index

##### Adverse reactions

**Fig. 15:**
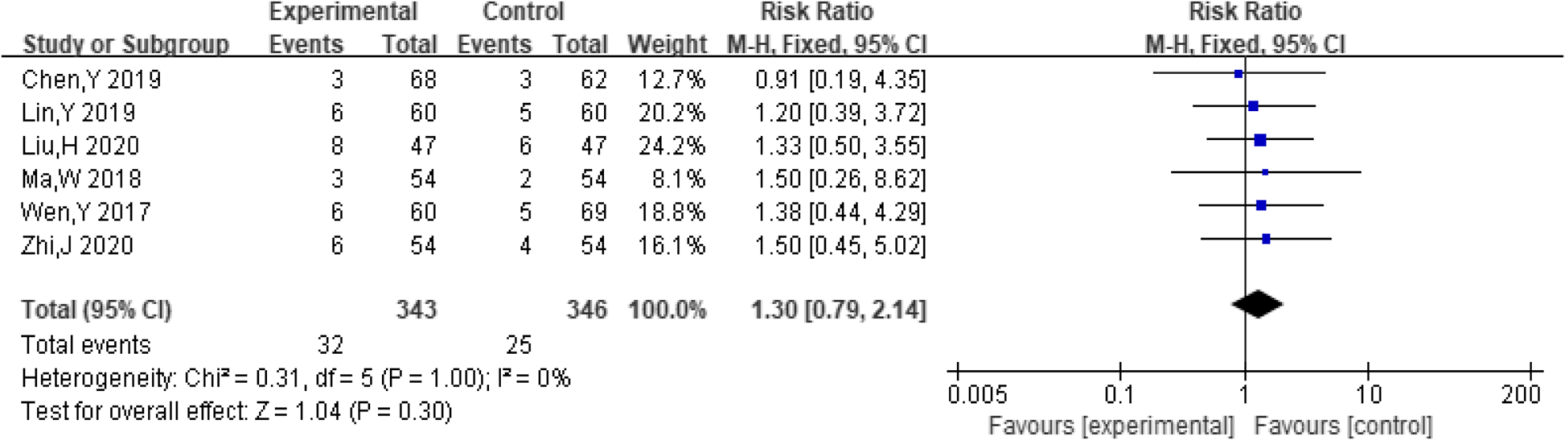
Forest plot of adverse reactions in patients with PCOS treated with dexamethasone combined with clomiphene

A total of six studies reported the number of people who experienced adverse effects. The pooling data resulted in no heterogeneity among the studies (I^2^ = 0%, *P* = 1.00). Therefore, the fixed effect model was chosen to analyze this outcome. The results showed that dexamethasone combined with clomiphene had insignificant effect on the incidence of adverse reactions in PCOS patients (*RR* = 1.30, 95% CI: 0.79, 2.14, Fig. 15).

##### Vertigo

**Fig. 16:**
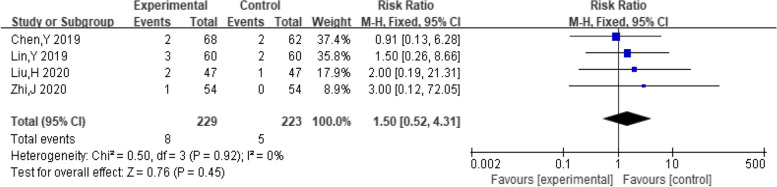
Forest plot of vertigo in patients with PCOS treated with dexamethasone combined with clomiphene

A total of six studies included the number of people who experienced vertigo. The pooling data resulted in no heterogeneity among the studies (I^2^ = 0%, *P* = 0.92). Hence, the fixed-effect model was selected for analyzing this outcome. The findings indicated that combination of dexamethasone and clomiphene had insignificant effect on the incidence of vertigo in PCOS patients (*RR* = 1.50, 95% CI: 0.52, 4.31, Fig. 16).

##### Vomiting

**Fig. 17:**
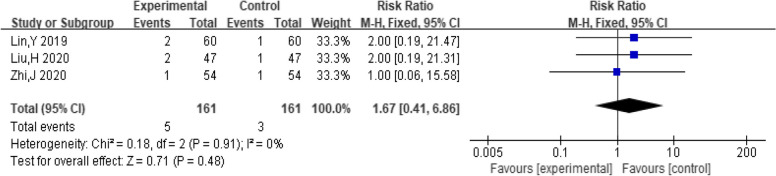
Forest plot of vomiting in PCOS patients treated with dexamethasone combined with clomiphene

A total of three studies included the number of people who vomited. The pooling data resulted in no heterogeneity among the studies (*I*^*2*^ = 0%, *P* = 0.91). Hence, the fixed-effect model was selected for analyzing this outcome. The results showed that the treatment of dexamethasone combined with clomiphene had insignificant effect on the incidence of vomiting in PCOS patients (*RR* = 1.67, 95% CI: 0.41, 6.86, Fig. 17).

### Sensitivity analysis

In this study, single-factor sensitivity analysis was conducted on the substantially heterogeneous outcomes including LH, ovulation-stimulating hormone, estradiol, progesterone, and T levels. After excluding any one study, the combined effects were relatively close than before, which suggested that the results of this study were stable.

## Discussion

Meta-analysis of this study showed that dexamethasone combined with clomiphene treatment significantly increased pregnancy and ovulation rates in patients with PCOS. This finding was consistent with the known role of dexamethasone in endocrine modulation and lowering of serum androgen levels, which was essential for improving ovarian response in patients with PCOS [21]. In particular, dexamethasone, by reducing testosterone levels in the ovaries, may have enhanced follicular responsiveness to clomiphene, thereby improving ovulation rates [18]. In addition, the treatment group showed significant improvements in luteinising hormone, oestradiol, progesterone and testosterone levels, and supported the ability of dexamethasone regulated hormonal balance, especially in reducing hyperandrogenaemia in patients with PCOS [19]. Decreases in luteinising hormone (LH) might directly affect follicular maturation and corpus luteum function, whereas improved progesterone levels indicated a better luteal phase response. On the other hand, an increase in oestradiolreflected follicular growth and maturation.It is a key prerequisite for ovulation.

Although our analyses showed these positive effects, the heterogeneity of the studies suggested a complex context. For example, the high heterogeneity in luteinising hormone and oestradiol levels might stem from differences in the dose of dexamethasone used and the duration of treatment in different studies. It is suggested that individualised treatment regimens may be even more important in practical clinical applications. Requiring patient-specific adjustments of drug dosage and treatment cycles is warranted. Furthermore, despite the low risk of publication bias, our research was limited by a small sample size and variations in the quality of the studies included, which may have a negative effect on the generalizability and reproducibility of our findings. In addition, although the results showed that the combination of dexamethasone and clomiphene, did not increase the risk of adverse effects, inconsistencies in monitoring and reporting of adverse events might mask potential safety concerns.

In summary, the treatment regimen of dexamethasone in combination with clomiphene shows clear potential for improving pregnancy and ovulation rates in patients with PCOS. Larger sample sizes, higher-quality RCTs to validate these findings and to explore how to make the most use of this combination therapy, including determining the optimal dosage and duration of treatment, as well as assessing long-term safety and efficacy of treatment is warranted in the future. In addition, given the multifactorial etiology of PCOS, exploring the combination of dexamethasone and clomiphene with other therapies such as insulin sensitivity improvers may provide a more comprehensive strategy for treatment.

## Data Availability

No datasets were generated or analysed during the current study.
